# The Glycan Role in the Glycopeptide Immunogenicity Revealed by Atomistic Simulations and Spectroscopic Experiments on the Multiple Sclerosis Biomarker CSF114(Glc)

**DOI:** 10.1038/srep09200

**Published:** 2015-03-17

**Authors:** Agostino Bruno, Mario Scrima, Ettore Novellino, Gerardino D'Errico, Anna Maria D'Ursi, Vittorio Limongelli

**Affiliations:** 1 Department of Pharmacy, University of Naples “Federico II”, via D. Montesano 49, I-80131 Naples, Italy; 2 Department of Pharmacy, University of Salerno, Via Giovanni Paolo II 132, I-84084 Fisciano, Italy; 3 Dipartimento di Scienze Chimiche, Università di Napoli “Federico II”, Complesso di Monte Sant'Angelo, via Cinthia, 80126 Naples, Italy; 4 Università della Svizzera Italiana (USI), Faculty of Informatics, Institute of Computational Science, via G. Buffi 13, CH-6900 Lugano, Switzerland

## Abstract

Glycoproteins are often recognized as not-self molecules by antibodies triggering the onset of severe autoimmune diseases such as Multiple Sclerosis (MS). Thus, the development of antigen-mimicking biomarkers represents an attractive strategy for an early diagnosis of the disease. An example is the synthetic glycopeptide CSF114(Glc), which was designed and tested as MS biomarker and whose clinical application was limited by its reduced ability to detect autoantibodies in MS patients. In the attempt to improve the efficacy of CSF114(Glc), we have characterized all the events leading to the final binding of the biomarker to the autoantibody using atomistic simulations, ESR and NMR experiments. The glycosydic moiety plays a primary role in the whole process. In particular, in an environment mimicking that used in the clinical tests the glycopeptide assumes a α-helix structure that is functional for the interaction with the antibody. In this conformation CSF114(Glc) binds the monoclonal antibody mAb8-18C5 similarly to the myelin oligodendrocyte glycoprotein MOG, which is a known MS auto-antigen, thus explaining its diagnostic activity. Our study offers new molecular bases to design more effective biomarkers and provides a most valid protocol to investigate other systems where the environment effect is determinant for the biological activity.

Posttranslational modification of proteins is an ingenious mechanism of the cell to differentiate and regulate the biological response. The major example is glycosylation, which plays a key role in controlling prokaryote and eukaryote cellular mechanisms^1^,^2^. In particular, the glycan added to a protein influences its molecular interactions in the cellular matrix, controlling important processes like cell adhesion, macromolecule interaction, and pathogen infections^1^,^3^,^4^. Furthermore, glycoproteins are involved in aberrant cellular mechanisms such as inflammatory processes, cancer and immune-mediated responses. In the latter case, the binding interaction between glycosylated proteins and antibodies is generally recognized as the onset mechanism of severe autoimmune diseases such as rheumatoid arthritis (RA)^5^,^6^, systemic lupus erythrematosus (SLE)^7^, and multiple sclerosis (MS)^8^. Thus, designing chemical probes able to detect autoantibodies represents an attractive strategy to develop diagnostic biomarkers^9^,^10^,^11^,^12^. A successful example is the MS biomarker CSF114(Glc) (Fig. 1a). This 21 aminoacids glycopeptide was indeed designed by some of us to mimic the sequence and the conformation of a portion (aa 30–50) of the human myelin oligodendrocyte glycoprotein (MOG), which is a MS auto-antigen. The rationale of the design is based on the finding that the portion of the glycoprotein which includes the 30–50 aminoacids sequence and the presence of the N-glucosylated asparagine at position 31 are responsible for the MOG antigenic activity^13^. CSF114(Glc) was found to bind different types of demyelinating autoantibodies (autoAb) in the sera of MS patients, however its clinical application was limited due to its reduced efficacy in detecting MS patients in the reported tests^14^. Thus, many efforts have been made to improve its antigenic properties by modifying its chemical structure, without achieving the desired results^14^,^15^.

In this framework, the elucidation of the CSF114(Glc) mechanism of action is of paramount importance to guide in a rational way the design of new biomarkers. Unfortunately, the binding interaction between a glycopeptide and its antibody is a complex process, which is regulated by a number of factors like the glycoprotein conformational flexibility and the environment effect^16^,^17^. These aspects limit the structural characterization of the binding mechanism using both theoretical and experimental techniques, thus hampering the rational design of new probes^11^,^12^,^18^,^19^,^20^,^21^,^22^. In the case of CSF114(Glc), NMR studies showed indeed a high conformational polymorphism of this glycopeptide, which assumes different conformations in diverse environments^14^,^15^. In particular, CSF114(Glc) adopts a β-hairpin-like conformation in water/HFA^14^, while it assumes a α-helix structure in the micelle environment^15^. These data indicate that the environment is able to stabilize specific conformations of the glycopeptide and understanding which one is competent for the activity is not an easy task. Previous studies show that the binding between CSF114(Glc) and autoAb occurs in a lipophilic environment^15^, which mimics the extracellular surface of the cellular membrane where the interaction between MOG and autoAb physiologically takes place^23^,^24^. In this paradigm, the elucidation of the binding mechanism of CSF114(Glc) to the cellular membrane is biologically of great relevance since it can provide the structural basis for the interaction with autoAbs. Thus, we decided to investigate this mechanism in details performing a series of calculations and experiments that allowed characterizing with atomistic resolution the molecular interaction of CSF114, in its glycosylated [CSF114(Glc)] and non-glycosylated (CSF114) form, with the membrane bilayer.

First, we performed 1 μs molecular dynamics calculations for each system, CSF114(Glc) and CSF114, using an advanced sampling approach, recently developed by some of us^25^. These simulations allowed describing the whole binding process of the glycopeptides to membrane, elucidating the interactions established between lipids, sugar and aminoacids. In particular, we found two different binding modes of CSF114(Glc) to the membrane. In the first one the glycosylated residue (Asn-Glc) engages strong interactions with the phospholipids polar heads, while in the second one the Asn-Glc moiety is exposed towards the solvent. At variance with CSF114(Glc), CSF114 establishes more superficial and transitory interactions with the membrane. To validate experimentally our *in-silico* model, we performed a series of Electron Spin Resonance (ESR) and NMR measurements. These results support our predictions showing that CSF114(Glc) and CSF114 differently interact with the membrane. In particular, the ESR spectra reveal that the glycosylated peptide binds more deeply the membrane with respect to the non-glycosylated one, while the NMR data confirm the binding mechanism of CSF114(Glc) to the membrane found in our simulations. The obtained structural information has been finally used to elucidate the binding mode of CSF114(Glc) to a monoclonal demyelinating antibody, namely mAb8-18C5^23^,^24^. This detailed description of the events not only paves the way to develop new MS biomarkers, but also it opens new areas of investigation for other biologically relevant systems, such as the HIV-1 membrane-proximal external region protein MPER^26^, where the protein/membrane interaction is a determinant factor.

## Results

### The CSF114(Glc)/membrane interaction

**Figure 1:**
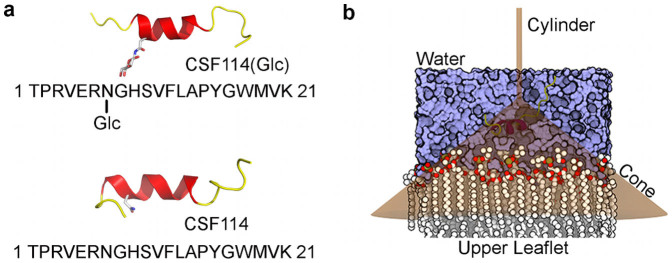
The CSF114(Glc) and CSF114 peptides. (a) Cartoon representation of the CSF114(Glc) and CSF114 peptides, together with the full length sequence of the peptides. (b) Cartoon representation of the funnel restraint applied to the CSF114(Glc)/membrane and CSF114/membrane systems.

**Figure 2:**
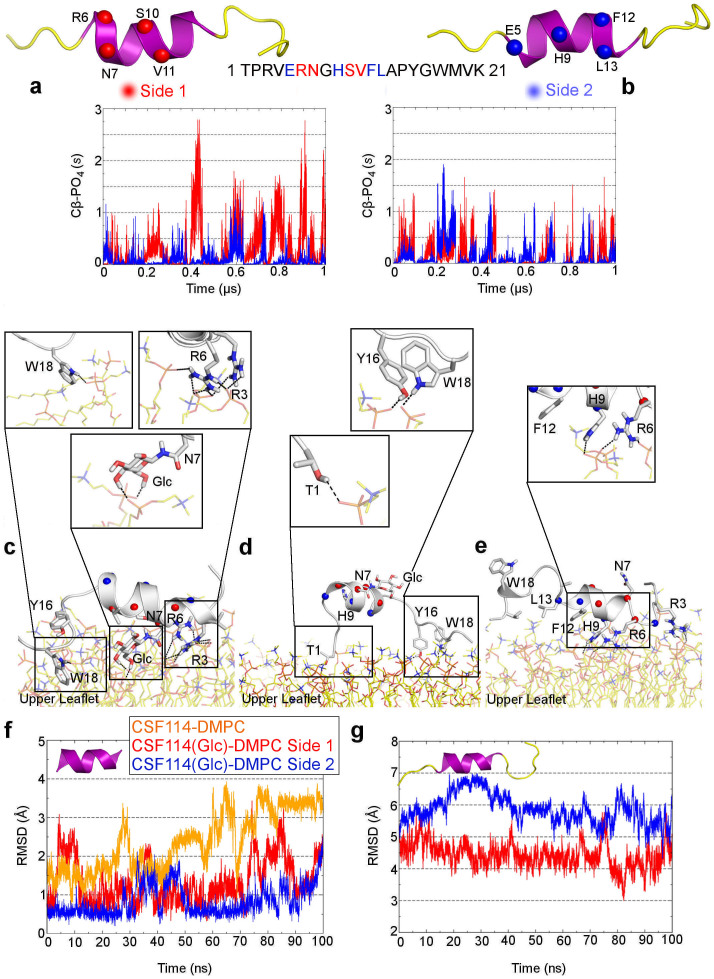
The CSF114(Glc) and CSF114 binding modes to the membrane. (a) and (b) Values of the contact collective variable computed between the membrane PO_4_ groups and the Cα atoms of CSF114(Glc) and CSF114, respectively. Two opposite sides of the α-helix structure of CSF114(Glc) and CSF114, Side 1 and Side 2, were considered in the analysis (see Methods for details). (c) and (d) Representation of the binding modes of CSF114(Glc) to the membrane resulting as the most populated conformational families from the cluster analysis (see Methods for details). Two different binding modes are found with the peptide interacting with the membrane through two opposite sides of the α-helix, Side 1 and Side 2 (c and d, respectively). The main peptide/membrane interactions are highlighted as inset. (e) Representation of the binding mode of CSF114 to the membrane. In this case, one highly populated conformational family was found in the cluster analysis (see Methods for details). The main peptide/membrane interactions are highlighted as inset. (f) Plot of the rmsd values of the Cα atoms of the core residues (5–12 aa) forming the α-helix structure computed respect to the NMR conformations. (g) Plot of the rmsd values of the Cα atoms considering all the residues, including the flexible N-ter and C-ter regions, computed respect to the NMR conformations.

The use of atomistic simulations represents a natural choice to investigate the binding process between two molecules. However, the binding event is a long timescale process, from μs to ms, which is difficulty described using standard simulations such as molecular dynamics (MD). Thus, to obtain a comprehensive description of the binding event one should step up the computational strategy. In this framework, we have recently developed an approach based on the use of a funnel-restrained potential that reduces the conformational space to explore in the unbound state, thus enhancing the binding events and allowing to describe the whole binding process in an affordable computational time^25^. In the present case, taking advantage of this approach we performed a series of funnel molecular dynamics simulations (FMD) in explicit solvent to investigate the molecular interactions formed by the CSF114(Glc) and CSF114 peptides in membrane environment, which is represented by a 49 nm^2^ bilayer of 1,2-dimyristoyl-*sn*-glycero-3-phosphocholine (DMPC) (Fig. 1b). Since NMR studies showed that these peptides have a α-helix structure in membrane-mimicking environment (see the “NMR spectroscopy” paragraph and Ref. 15), we decided to preserve this conformation for both peptides during these simulations (see Methods for details). For each peptide, in 1 μs of FMD calculations several binding events to the membrane were observed. The atomistic interactions engaged by the peptides with the phospholipid bilayer were characterized and assessed using a contact collective variable (*s*) (See Methods). In particular, we found that both peptides interact with the membrane through two opposite sides of the α-helix (side 1 and 2 in Fig. 2a–b). Looking at Fig. 2a and 2b, one can note that CSF114(Glc) shows a higher number of contacts with the phospholipids than CSF114. Furthermore, as regards CSF114(Glc) the side presenting the glycosylated residue (Side 1) forms a higher number of interactions and for longer time than the non-glycosylated one (Side 2) (Fig. 2a). At variance with CSF114(Glc), CSF114 establishes more superficial and transitory contacts with the phospholipids (Fig. 2b), suggesting that the glycosylated residue (Asn-Glc) plays an important role in the binding to the membrane.

Movies showing the binding of CSF114(Glc) and CSF114 to the membrane under the action of FMD simulations are provided as Supplementary Movies.

Prompted by these results, we decided to characterize at atomistic resolution the interactions engaged by CSF114(Glc) and CSF114 with the phospholipid bilayer. A detailed discussion is reported in the following paragraphs.

### The CSF114(Glc) binding modes to membrane

For both CSF114(Glc) and CSF114 we considered as bound state all the conformations obtained from the FMD simulations that has the contact value *s* higher than 0.5 (See Fig. 2a, 2b and Methods for details). All these states were clustered in families according to their binding conformation (see Methods for details) and the representative poses of the most populated clusters are shown in Fig. 2c–e. As regards CSF114(Glc), two different binding modes were found where the peptide interacts with the membrane through two opposite sides of the α-helix structure (Fig. 2c and 2d). In the first one, which represents the most populated family, the peptide binds deeply the membrane through its glycosylated side. In the other binding mode, the peptide/membrane interaction is more superficial with the glycosylated side pointing toward the solvent.

In particular, in the first binding mode CSF114(Glc) engages with the phospholipids a large number of interactions through residues such as Arg^3^, Arg^6^, Trp^18^, Asn^7^ and the glycosyl moiety (Fig. 2c). In fact, Arg^3^ and Arg^6^ form salt bridge interactions with the membrane phosphate groups, while Trp^18^ is placed between the positively charged nitrogens of two choline groups, where it can engage cation-π interactions. Furthermore, the glycosyl moiety establishes H-bond interactions with the membrane phosphate groups, contributing to further stabilize this pose. In the second binding mode CSF114(Glc) interacts with the membrane through different residues such as Thr^1^, Tyr^16^ and Trp^18^. In this pose, the side chains of these residues form H-bond interactions with the membrane phosphate groups (Fig. 2d). It is interesting to note that in both the binding modes the flexible C-ter region (Tyr^16^-Lys^21^) interacts with the membrane (Fig. 2c-e), suggesting a functional role played by these residues during the binding event. This finding explains our previously reported results showing that the presence of the C-ter region is fundamental for the antigenic activity of CSF114(Glc) in antibody binding assays^14^,^15^.

As regards CSF114, only one highly populated family was found in the cluster analysis. In this pose, CSF114 establishes a smaller number of contacts with the membrane with respect to CSF114(Glc) (Fig. 2b and 2e). In particular, Arg^3^, Arg^6^ and His^9^ form salt bridge and H-bond interactions with the membrane phosphate groups, while Phe^12^ is close to the positively charged choline groups, where it can engage cation-π interactions (Fig. 2e).

To assess the stability of the identified binding conformations we performed an over 100 ns long MD simulation for each binding mode of CSF114(Glc) and CSF114 (see Methods and Supplementary Information for details). At variance with the FMD calculations, in these simulations we allowed full flexibility to the two peptides. Both of them show a good conformational stability of the α-helix in all the simulations, as demonstrated by the low rmsd values computed for the backbone atoms of the core residues forming the α-helix structure (Fig. 2f). Furthermore, the two different binding modes of CSF114(Glc) are equally stable conserving all the previously described glycopeptide/membrane interactions throughout the whole calculation (see Fig. 2c, d and Supplementary Fig. S1). However, the N-ter and C-ter regions are rather flexible as demonstrated by the higher rmsd values computed for the peptide backbone atoms including also these residues (Fig. 2g) if compared with those computed considering only the core residues forming the α-helix structure (Fig. 2f). All the states obtained from these MD simulations were clustered in families and the representative conformation of each family was used in the subsequent docking study (see Methods and “The CSF114(Glc)/antibody complex” paragraph).

A movie showing the stability of CSF114(Glc) in the two binding modes to the membrane is provided as Supplementary Movies.

At variance with CSF114(Glc), the CSF114 binding mode is less stable during the 100 ns long MD simulation, due to the weaker interactions engaged with the membrane (see Fig. 2f and Supplementary Fig. S1).

To investigate the effect of the membrane on the α-helix structural stability of CSF114(Glc) and CSF114, we performed additional MD simulations on both the peptides in fully water systems. In both cases, the α-helix structure is unstable and is completely lost in the first 10 ns of simulation (See Supplementary Fig. S2). This result suggests that the membrane environment favours the α-helix stability as also reported by experiments^15^. Further details on these simulations are discussed in the Supplementary Information.

### The environment effect

**Figure 3:**
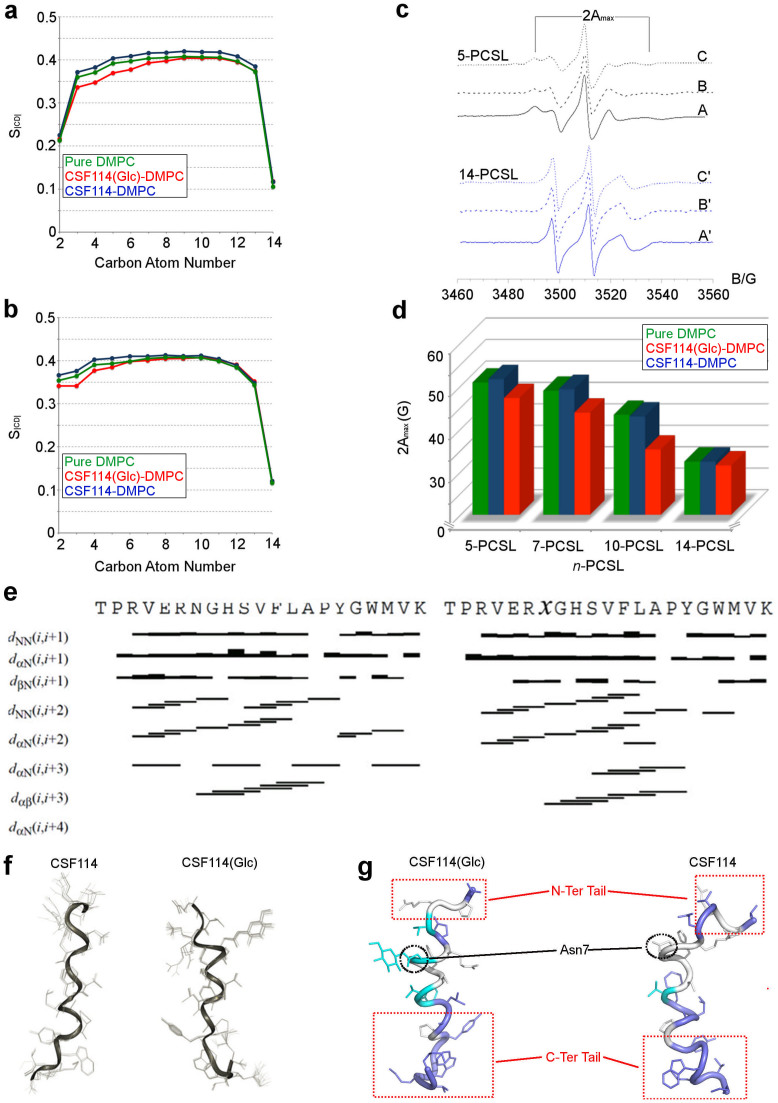
Structural basis for the peptide/membrane interaction. (a) and (b) Plots of the S_|CD| _parameter computed for the c1 and c2 chains, respectively, of the DMPC phospholipids along the binding simulations. (c) ESR spectra of *n*-PCSL (*n* = 5,14) positional isomers in DMPC membranes in the absence (A,A′) and in the presence of CSF114 (B,B′) or CSF114(Glc) (C,C′). (d) Dependence on spin-label position, *n*, of the outer hyperfine splitting, coupling constant 2A_max_, in the absence (green bars) and in the presence of CSF114 (blue bars) or CSF114(Glc) (red bars) (The Standard errors are reported in Supplementary Table 1). (e) NOE connectivities collected in NOESY spectra of CSF114 (left) and CSF114 (Glc) (right) in DPC micelle solution. NOESY spectra were acquired at 600 MHz 300 k. (f) Representation of the NMR structure bundles of CSF114 and CSF114(Glc) in DPC micelles. The 20 best structures were overlapped on the backbone atoms showing very low rmsd values of 0.09 Å and 0.08 Å for CSF114 and CSF114(Glc), respectively. (g) The Lowest energy NMR structures of CSF114(Glc) (left) and CSF114 (right) in DPC micelles. Color blue, light blue and white indicate residues with strong, medium and no attenuation of the NMR signals in the presence of *5- doxilsteric acid*, respectively.

In line with the experimental evidences (see the “NMR spectroscopy” paragraph and Ref. 15), our simulations indicate that the membrane environment favours the α-helix conformation of CSF114(Glc) and CSF114 (see Supplementary Information). This structure is stabilized by several interactions engaged between the phospholipids and the aminoacid residues, particularly in the case of CSF114(Glc). We decided to assess these interactions calculating the order distribution parameter of the bilayer (S_CD_) during the binding of the two peptides. This variable estimates the organization of the lipids forming the membrane. In particular, higher is the S_CD_ value, greater is the order degree of the lipids and more compact is the membrane (See Methods for details). The parameter S_CD_ was computed along the MD simulations of CSF114(Glc) and CSF114 bound to the membrane and during an MD simulation of the bilayer in absence of peptides that was used as reference system (Fig. 3a and 3b). In the latter case, it is worth noting that the calculated S_CD_ values are in agreement with the experimental and computational data reported in literature^27^. As regards CSF114(Glc) and CSF114, both peptides have effect on the organization of the lipids, particularly in the external part of the membrane (Fig. 3). In fact, both peptides induce conformational changes of the bilayer from the polar head to the twelfth carbon atom of the c1 chain and to the seventh carbon atom of the c2 chain of DMPC (Fig. 3a and 3b, respectively). Furthermore, comparing Fig. 3a and 3b one can note that the glycosylated peptide affects mostly the organization of the membrane than the non-glycosylated one. These data provide a further evidence of the stronger and deeper interaction with the membrane of CSF114(Glc) with respect to CSF114. The latter peptide shows indeed S_CD_ values close to those computed in the pure DMPC system, indicating a more superficial interaction of CSF114 with the membrane.

### Electron Spin Resonance (ESR)

To validate our binding models we performed a series of experiments. In particular, Electron Spin Resonance (ESR) spectroscopy was carried out at similar conditions to our simulations. First, DMPC membranes incorporating phosphatidylcholine spin-labeled on the 5 C-atom of the sn-2 chain (5-PCSL) were investigated. This label presents the nitroxide group close to its hydrophilic head group, thus representing a good probe to assess superficial effects. The ESR spectrum of 5-PCSL in DMPC membranes, shown in Fig. 3c (black solid line), presents a clearly defined axially anisotropic lineshape, with a value of the outer hyperfine splitting, 2A_max_, equal to 49.6 ± 0.1 G. We also investigated DMPC membranes including phosphatidylcholine spin-labeled on the 7,10 and 14 C-atom of the sn-2 chain (7-, 10- and 14-PCSL, respectively). In particular, for the 14-PCSL the nitroxide group is positioned close to the terminal methyl region of the chain. In this case, the label will provide information about the effect of the peptides binding to the inner region of the membrane. For DMPC membranes in absence of peptides a narrow three-line, quasi-isotropic spectrum is obtained (Fig. 3c, blue solid line). The higher isotropy of the 14-PCSL spectrum with respect to that obtained for 5-PCSL indicates a flexibility increase in segmental chain mobility in going from the polar head groups to the inner hydrophobic core, which is a characteristic hallmark of the liquid-crystalline state of fluid phospholipid bilayers^28^.

The association of the two peptides [CSF114 and CSF114(Glc)] with lipid membranes was detected by the perturbation of the chain mobility of spin-labeled lipids, by using ESR spectroscopy as found for classical water-soluble peripheral membrane proteins and derived peptides^29^,^30^,^31^. Association of peptides to the lipid bilayer causes a significant variation in the ESR spectra of spin-labeled phospholipids. As shown in Fig. 3c, the presence of two peptides induces significant changes in the spin-label ESR spectra, which are mainly detectable from the low- and high-field component position and line shape. In the attempt to quantify this evidence, the 2A_max_ values for pure DMPC bilayer and in presence of CSF114 and CSF114(Glc) were determined and reported in Fig. 3d (In Supplementary Table 1 the standard errors are also reported). It is possible to note that, in all cases, the flexibility gradient with the chain position of the lipid bilayer membranes is preserved. However, inspection of Fig. 3d reveals a significantly different behavior of the lipid chain mobility depending on the presence of the two peptides.

In particular, addition of CSF114 peptide causes an increase of anisotropy of 5-, 7- and 10-PCSL, as also demonstrated by the increase in the 2A_max_ values (Fig. 3d), while no change in the spectra of the spin-label bearing the nitroxide group in the more interior position (14-PCSL) was observed. On the contrary, addition of the CSF114(Glc) peptide induces a strong decrease of the 2A_max_ values for all the considered spin-labels (Fig. 3d). These findings indicate that CSF114(Glc) interacts more strongly and deeply with the membrane than CSF114, in line with our *in-silico* model. In particular, CSF114 interacts with the lipid bilayer causing a slight perturbation, which does not involve the inner part of the membrane. At variance with CSF114, CSF114(Glc) causes a stronger destabilization of the bilayer microstructure and the increase of segmental mobility propagates along the whole acyl chains.

### NMR spectroscopy

Determination of NMR structure for short peptides is generally hampered by their flexibility. In these cases, indeed, NMR “structure” only corresponds to conformational ensembles reflecting the peptide conformational preferences. Nevertheless, NMR spectroscopy allows peptide conformational analysis in conditions that may closely mimic the biological environments where the peptide is active. On this basis, in view of finding experimental support to our MD and EPR data showing that the membrane interacts with the CSF114(Glc) peptide stabilizing its binding conformation, we undertook an extensive NMR investigation of CSF114(Glc) in DPC micelle solution. This solvent was chosen as a membrane mimetic system (Fig. 3e, 3f). 2D ^1^H TOCSY and NOESY experiments were recorded on NMR 600 MHz. Spectral assignment was achieved by analysing experiments according to Wüthich's procedure^32^. Using CYANA software, on the basis of NOE data, conformational ensembles of CSF114(Glc) and CSF114 were obtained (Fig. 3e, 3f). According to the collected short and medium range NOE connectivities, in both peptides regular secondary structures are evident in the central portion of the sequence (Arg^6^-Ala^14^, Fig. 3e). Fig. 3f shows the NMR structure bundles of CSF114 and CSF114(Glc), which result to be highly defined with very low rmsd values of 0.09 Å and 0.08 Å computed for the backbone atoms of CSF114 and CSF114(Glc), respectively. Analysis of backbone dihedral angles indicates for both the peptides the presence of nascent helical structures in the central part of the sequences (Arg^6^-Ala^14^, Fig. 3e, 3f), with the non-glycosylated one being less structured. These findings are in agreement with the NMR data on CSF114(Glc) previously reported by some of us^15^. On the other hand, short and medium range NOE connectivities show the presence of unstructured region at the N-ter and C-ter of CSF114(Glc) (Thr^1^-Val^4^ and Pro^15^-Lys^21^, respectively), denoting a high flexibility of these residue in agreement with our MD results.

To investigate the positioning of the peptide with respect to the surface of the micelle, we recorded NMR TOCSY experiments in presence of 5-doxyl-stearic acid paramagnetic probe. This molecule is a spin label used in Paramagnetic Relaxation Enhancement (PRE) to investigate structural changes in lipids since it presents an unpaired electron on the doxyl group bound to the aliphatic chain carbon in position 5 of the stearic acid^33^. Unpaired electrons lead to a strong acceleration of the longitudinal and transverse relaxation rates of protons in spatial proximity via highly efficient spin-electron relaxation. This is a long-range effect, in the order of 15–24 Å from the labeled atom. As a result, the paramagnetic probe induces a broadening of the NMR signals and the decrease of resonance intensities for residues close to the surface (5-doxyl) of the micelle. Thus, using such technique one can probe chemical local environment obtaining long-range distance information which complements that coming from NOE restraints, which are limited to distances of less than 6 Å. The TOCSY spectra of CSF114(Glc) and CSF114 reveal a clear tendency of the peptides to be in contact with the micelle compartment (Fig. 3g and Supplementary Table 2). In particular, we found a significant reduction in intensity of NH signals of Thr^1^, Glu^5^, and Tyr^13^-Lys^21^ in both the peptides, thus denoting the involvement of N-ter and particularly C-ter residues in the binding to the membrane (Fig. 3g and Supplementary Table 2). The two peptides show instead a different interaction with the micellar surface in the central region of the α-helix structure. In fact, the signal belonging to the NH of Asn^7^ is perturbed only in CSF114(Glc), suggesting that Asn^7^ interacts with the membrane solely in its glycosylated form. These experimental observations agree with the previously reported binding modes showing the key role played by the glicosylated Asn^7^ residue in the binding to the membrane (Fig. 2c–e).

### The CSF114(Glc)/antibody complex

**Figure 4:**
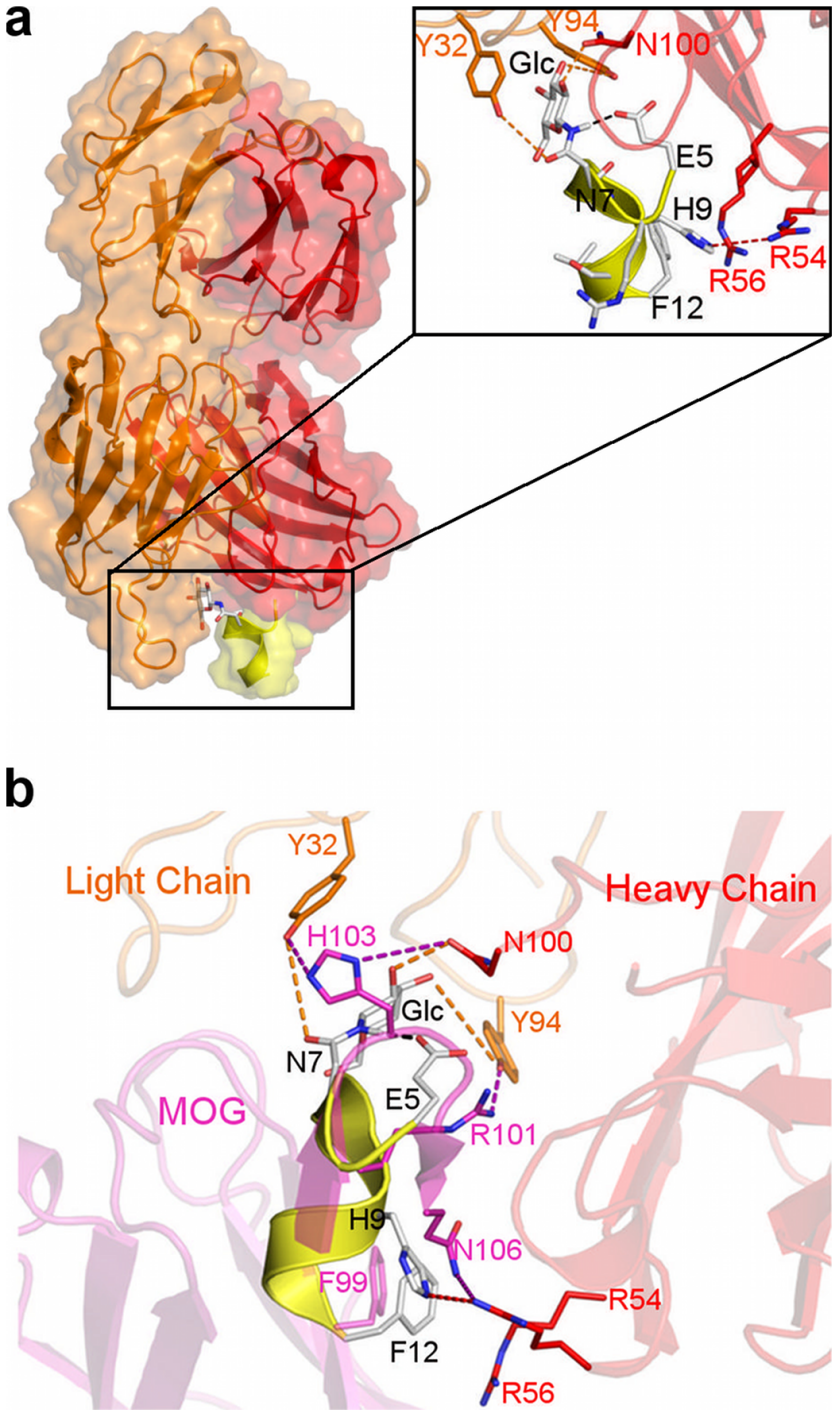
The CSF114(Glc)/mAb8-18C5 complex. (a) Representation of the binding mode of CSF114(Glc) to the monoclonal demyelinating antibody mAb8-18C5 obtained from docking calculations. The CSF114(Glc) peptide is shown in yellow while the light and heavy chains of autoAb are colored in orange and red, respectively. In the inset, residues of CSF114(Glc), autoAb light and heavy chain, are shown as white, orange and red sticks, respectively. (b) Superimposition of the computed binding mode of CSF114(Glc) on the X-ray crystal structure of the MOG/mAb8-18C5 complex. The CSF114(Glc) and mAb8-18C5 residues are represented according to the above described color code, while MOG is shown in purple.

Once we disclosed the binding mechanism of CSF114(Glc) to the membrane, we decided to investigate also its molecular interaction with the monoclonal antibody mAb8-18C5. This antibody binds the MOG peptide inducing the demyelination of neurons in myelin-containing tissue^23^,^24^,^34^. Furthermore, mAb8-18C5 is the only antibody whose structure has been resolved through X-ray in complex with the MOG peptide (PDB code 1PKQ). Specifically, the representative conformation of each cluster family obtained from the previously discussed MD simulations on the two binding modes, Side 1 and Side 2 (see Supplementary Fig. S3), was docked in the X-ray complementary-determining regions (CDRs) of mAb8-18C5^23^,^24^. This is indeed the site where antigens are typically bound (see Methods for details). This ensemble docking protocol allows considering during the calculation a large number of CSF114(Glc) conformations, overcoming the limitation of the docking algorithm that treats the protein as a rigid body. Due to the high conformational flexibility of the N-ter and C-ter region, only the core residues (5–12) forming the α-helix structure of CSF114(Glc) was considered in the docking calculations. In one of the best-scored poses, CSF114(Glc) engages a number of favorable interactions with the antibody CDRs (Fig. 4). In particular, Asn^7^ of CSF114(Glc) forms H-bonds with Tyr32 and Tyr94 of the antibody light chain, while its glycosyl moiety H-bonds with Asn100 of the heavy chain. Furthermore, His^9^ of CSF114(Glc) engages H-bond interactions with Arg54 of the heavy chain, while Phe^12^ establishes a cation-π interaction with Arg56 that further stabilizes the complex.

Comparing the CSF114(Glc) binding mode with that of the MOG peptide resolved by X-ray (PDB code 1PKQ), a common pattern of interaction can be found (Fig. 4b). In particular, in the MOG/mAb8-18C5 complex His103 and Arg101 of MOG form H-bonds with Tyr32, Tyr94 and Asn100 of the antibody. Similar interactions are engaged by the glycosylated residue Asn^7^(Glc) in the CSF114(Glc)/mAb8-18C5 binding complex. Furthermore, Asn106 and Phe99 of MOG interact with Arg54 and Arg56, respectively, as similarly done by His^9^ and Phe^12^ of CSF114(Glc) (Fig. 4b).

Looking at the CSF114(Glc) binding mode (Fig. 4a), one can note the presence of an intramolecular H-bond between the side chains of Glu^5^ and Asn^7^(Glc). This interaction is important to stabilize the CSF114(Glc) conformation competent for the binding to the antibody. In fact, experimental evidences have shown that the mutation of Asn^7^(Glc) in Ser(Glc), which cannot form the intramolecular H-bond, decreases the probe activity^35^,^36^. Furthermore, the presence of this intramolecular interaction blocks the glycosylated residue in the proper conformation to H-bond with residues important for the antibody binding such as Tyr32 and Asn100. In fact, the same residues engage H-bonds with MOG His103 in the MOG/mAb8-18C5 crystal structure and mutagenesis experiments have proven that these interactions are essential for the MOG binding to the demyelinating antibody^37^.

## Discussion

The development of biomarkers of severe pathologies such as multiple sclerosis (MS) is of primary importance to promptly start the therapy and ameliorate the life exaptation of patients. In this view, we successfully designed the glycopeptide CSF114(Glc)^14^, which is able to bind MS autoantibodies. However, its clinical use is limited by the low efficacy in detecting MS patients. Thus, it is of great value to elucidate the molecular interaction of CSF114(Glc) with MS antibodies to guide the design of more efficient biomarkers. To this end, the use of atomistic simulations represents a natural choice, however this task is made difficult by many factors like the glycoprotein conformational flexibility and the effect of the environment in which the binding between the probe and MS antibodies occurs^16^,^17^. Combining a series of simulations and experiments, we were able to consider in an accurate way all these factors shedding light on the functional mechanism of CSF114(Glc) as MS diagnostic probe.

First, we investigated the binding mechanism of this glycopeptide to a membrane bilayer. This is the very first event of binding since experimental evidences have shown that the interaction between CSF114(Glc) and MS antibodies occurs in a membrane-mimicking environment^14,15^. We have found that when the glycopeptide binds to membrane, it assumes a α-helix structure that is functional for the subsequent binding to the antibody. We demonstrate that the membrane environment favors the stability of this α-helix structure. Similar effects have been already reported in literature where the membrane promotes the formation of the bioactive conformation of small peptides^38^,^39^,^40^,^41^,^42^. In particular, CSF114(Glc) binds the membrane in two equally possible ways. In the first one, CSF114(Glc) interacts strongly with the membrane through its glycosylated part, engaging a number of interactions with the phospholipids that stabilize the bioactive α-helix structure. In the second binding mode, the peptide is rotated on the membrane surface exposing the glycosyl moiety towards the solvent.

**Figure 5:**
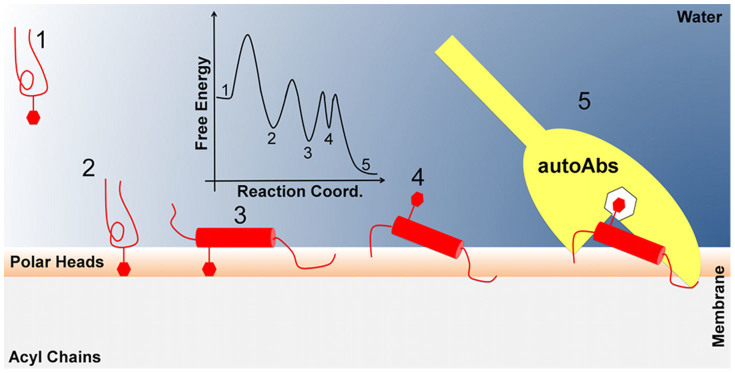
Cartoon representation of the functional mechanism of CSF114(Glc). Cartoon representation of the functional mechanism of CSF114(Glc). (1) From the bulk solvent CSF114(Glc) binds the membrane surface through its sugar moiety (red hexagon, 2). This interaction favors the structural organization of the glycopeptide in an α-helix structure (red cylinder, 3). Here, the peptide can rearrange in a conformation that exposes the Asn-Glc epitope on the membrane surface (4), thus promoting the interaction with the antibody (yellow, 5). The Artist's impression of the monodimensional free-energy profile of the whole process is represented as black line.

Based on our results, it is suggestive to propose a two-steps binding mechanism of CSF114(Glc) to the membrane. First, the peptide binds the bilayer through its glycosylated side, corresponding to the first binding mode (Fig. 2c), and then it changes into the second binding mode exposing the glycosylated side towards the solvent (Fig. 2d). In this state, CSF114(Glc) can present the Asn-Glc epitope to the antibody and functions as diagnostic probe (Fig. 5). This mechanism is further supported by docking calculations performed between CSF114(Glc) and the monoclonal demyelinating antibody mAb8-18C5^23^,^24^. In fact, we have found that CSF114(Glc) is able to bind the antibody only through its glycosylated side. In particular, it engages a number of favourable interactions with mAb8-18C5 through the glycosylated Asn^7^ and other residues such as His^9^ and Phe^12^. Furthermore, comparing the CSF114(Glc) binding mode with that of the glycoprotein MOG, which is a known MS auto-antigen, many similarities are found (Fig. 4b). In particular, the glycosyl residue engages interactions equivalent to those established by His103 of MOG, which is a fundamental residue for the binding of MOG to the antibody according to mutagenesis data^37^. Thus, preserving these interactions represents a good strategy in designing new MS antigenic probes.

In conclusion, our study sheds light on the functional mechanism of CSF114(Glc), illuminating the fundamental role played by the glycosylated residue during the binding to the membrane and the interaction with the antibody. Many experimental evidences, previously not explained, are here for the first time elucidated. For instance, mutagenesis experiments have shown that the mutation of Asn^7^(Glc) in Ser(Glc) decreases the probe activity^35^,^36^. According to our results, this residue is indeed involved in an intramolecular H-bond with Glu^5^ that stabilizes the bioactive conformation of CSF114(Glc). Its mutation in Ser(Glc) does not allow the formation of this intramolecular interaction, leading to a drop of the activity. Furthermore, our results provide useful hints to design new effective MS probes. For instance, we found that the C-ter region of CSF114(Glc) is involved in the binding to the membrane. The introduction of a palmitoyl moiety at C-ter could increase the number of interactions between the peptide and the phospholipids, thus enhancing the binding affinity to the membrane. Similar modifications have been indeed made to the MPER region of HIV gp41, leading to more immunogenic peptides^43^. Furthermore, the mutation of Gly^8^ in a polar residue, such as Lys, Arg or Gln, that is able to form interactions similar to those engaged by Arg101 of MOG (see Supplementary Fig. S4), might improve the binding affinity of CSF114(Glc) to the antibody and hence its diagnostic activity. However, we stress that different auto-antibodies are responsible for MS, thus the binding interaction of new probes should be however evaluated with a number of antibodies to increase the success rate of biomarker design.

Finally, our study and our approach are instrumental for investigations on the functional mechanism of other biologically relevant peptides, such as the HIV-1 membrane-proximal external region protein MPER^26^ and the N-glycans involved in metastatization process^19^, that are responsible for the onset of severe pathologies through their interaction with the cellular membrane.

## Methods

### Computational Protocol

#### System parameterization

The CSF114(Glc) starting structure was obtained from the NMR experiments described below and then parameterized using the *ff99SBildn*, *gaff*, and *GLYCAM_06* force fields of Amber11 suite^44^. The structure of the non-glycosylated peptide, CSF114, was obtained from the NMR experiments described below and then parameterized using the *ff99SBildn* force field. DMPC bilayer was built using the available parameters for Amber11 (http://www.pharmacy.manchester.ac.uk/bryce/amber#lip)^27^.

#### Systems setup and MD simulations

All the simulations were carried out using the NAMD 2.8 software^45^,^46^ and TIP3P as water solvation model^47^. For each peptide, CSF114(Glc) and CSF114, two systems were built: (*i*) peptides solvated in presence of the DMPC bilayer and (*ii*) peptides solvated in fully water.

#### Water/DMPC MD simulations

For the simulations in explicit DMPC membrane environment, we first built the DMPC-TIP3P system, DMPC bilayer was solvated using the vmd autoionize plugin^48^,^49^. The box dimension was equal to 86 × 86 × 110 Å, with a total amount of 76675 atoms. The DMPC-TIP3P system was equilibrated as follows: (*i*) 4000 steps of conjugate gradient minimization, where harmonic constraints on the system were progressively reduces; (*ii*) 90 ps of heating phase from 50 K to 300 K using the Langevin thermostat, where harmonic constraints on the lipid atoms were progressively reduces; (*iii*) 70 ps of pressure equilibration with a target pressure of 1.01325 bar and using the Nosè-Hoover Langevin barostat, where harmonic constraints were gradually switched off; (*iv*) 100 ps of pressure equilibration without constraints; (*v*) 100 ns of production phase. The production phase was carried out using the periodic boundary conditions in the NPT ensemble with a target pressure equals to 1.01325 bar and a surface tension target equals to 26 dyn/cm (as indicated in Ref. 27). The time-step was set to 2 fs, and the RATTLE algorithm was used for the hydrogen atoms. The temperature was retained at 300 K using the Langevin thermostat. A cutoff of 12 Å was set for both electrostatic and van der Waals short-range interactions, while the long-range electrostatics were treated using the PME methodology^50^ with a grid spacing of 1.0 Å and an interpolation order of 4.

#### Generation of the Peptide-Membrane complexes and FMD simulations

The peptide-DMPC-TIP3P complexes were built using as membrane bilayer that obtained from the last frame of the DMPC-TIP3P simulations, deleting all the water molecules. The initial distance between the center of mass of the peptide and the center of mass of the membrane was set equal to 35 Å. The peptide-DMPC systems were solvated using the vmd autoionize plugin^48^,^49^. The final box dimension for each system was equal to 70 × 70 × 155 Å. The obtained systems were submitted to a relaxation protocol as described in the *System Construction and MD simulations section* reported in the Supplementary Information. Finally, a production phase was conducted for 10 ns in the NPT ensemble with a target pressure equal to 1.01325 bar, a time-step of 2 fs, and using the RATTLE algorithm for the hydrogen atoms. The temperature was retained at 300 K using the Langevin thermostat. During the 10 ns of production phase, the position of the peptides relative to the membrane was restrained and the α-helix conformation was preserved using a harmonic constraint of 20 kcal/mol*Å^2^ on the peptide backbone atoms from residue Arg^6^ to residue Ala^14^. The last frames of the 10 ns simulation for each peptide-DMPC-TIP3P complex was used as starting structure to run 10 independent runs of FMD simulations (100 ns each one), reaching a total simulation time of 1 μs for each system. We stress that during the FMD simulations the α-helix structure for both CSF114(Glc) and CSF114 was conserved. This is possible since previously reported data^15^ and our NMR results (see the “NMR spectroscopy” paragraph) demonstrate that both the peptides have a α-helix structure in membrane-mimicking environment. The conformational space to explore was reduced using a funnel-restrained potential^25^. This potential is a combination of a cone restraint, which includes the external side of the membrane, and a cylindric part, which is directed toward the solvent. Following Ref. 25, we set the alpha angle to 0.8 rad, Rcyl to 1 Å and Zcc to 35 Å from the center of the membrane (See Supplementary Fig. S5). Using the funnel potential during the simulation, as the peptide reaches the edge of the funnel, a repulsive bias is applied to the system, disfavoring it from visiting regions outside the funnel^25^. Finally, the frames where the system feels the external potential were not considered for the statistical analysis of the simulations.

Methods and a detailed discussion of the simulations of the peptides in fully water systems are reported in Supplementary Information.

#### Analysis of the membrane-peptide contacts

We analyzed the number and type of contacts formed between the membrane and the peptides during the FMD simulations. To this end, we used a contact collective variable (*s*) defined by the following switching functions:
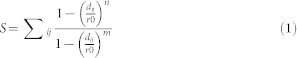
The parameters r_0_, n and m were set to 5, 8 and 16, respectively. Supplementary Table 3 lists the protein and membrane atoms considered for the contact collective variable. The analysis was performed using the PLUMED plugin^51^.

#### Membrane order parameters

The order parameters [S_CD_, equation (2)] is a direct measure of the relative distribution of the lipid chain into the bilayer^52^,^53^. The higher is the S_CD_ value, the greater will be the order of the lipid inside the membrane and more compact will be the membrane.

The relative order of the hydrocarbon tails can be obtained from S_CD_, where θ is the angle of a C−H vector with respect to the bilayer normal.
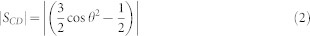


#### Cluster analysis

The binding mode of CSF114(Glc) and CSF114 to the membrane reported in Fig. 2C-E was obtained from a conformational cluster analysis of the FMD simulations. In details, only the states showing the contact collective variable *s* greater than 0.5 were considered as bound pose and hence clustered in families based on the rmsd values of the Cα atoms of the helix core (5–12 aa). The clustering was carried out using the ptraj tool^44^ and the average-linkage algorithm. The representative conformations of the most populated cluster families, which are the poses with the lowest rmsd value respect to the average structure of the clustered conformers population, are the binding modes of CSF114(Glc) and CSF114 reported in Fig. 2C–E.

A conformational cluster analysis was also performed for the MD simulations of the two CSF114(Glc)/membrane binding modes, Side 1 and Side 2. Even in this case the MD states were clustered based on the rmsd values of the Cα atoms of the helix core (5–12 aa). The clustering was carried out using the ptraj tool^44^ and the average-linkage algorithm. In this case, ten different families were obtained for each binding mode and the representative conformation of each family was considered in the docking calculations (see Supplementary Fig. S3).

#### Docking studies

CSF114(Glc) was docked in the X-ray crystal structure of mAb8-18C5 (PDB code 1PKQ)^24^ using the Glide suite of Maestro9.3.5^54^. The starting conformations of CSF114(Glc) used in the docking study were obtained from the cluster analysis of the MD simulations on the two CSF114(Glc)/membrane binding modes as above described. Due to the high conformational flexibility of the N-ter and C-ter region, only the core residues (5–12) forming the α-helix structure was considered in docking calculations. Thus, the peptide was neutralized at N-ter and C-ter, and geometrically optimized by means of Macromodel^54^ using the OPLS2005 force field in implicit solvent until a convergence value of 0.05kcal/mol*Å^2^. The mAb8-18C5 structure was prepared through the Protein Preparation Wizard of the Maestro9.3.5^54^. The grid box was centered on the complementary-determining regions (CDRs) of the light and heavy chains (See Supplementary Fig. S6). Each docking run was carried out with the standard precision (SP) method and the van deer Waals scaling factor of non-polar atoms was set to 0.8. The representative conformation of each family obtained from the above described cluster analysis was undergone to docking calculations, leading to a total of 20 docking calculations. The docking poses were finally ranked using the Glide Score scoring function^54^.

### ESR experiments

#### Materials

Dimyristoyl phosphatidylcholine (DMPC) was obtained from Avanti Polar Lipids (Alabaster, AL, USA). Spin-labeled phosphatidylcholine (1-acyl-2-[n-(4,4-dimethyloxazolidine-N-oxyl)]stearoyl-sn-glycero-3-phosphocholine, n-PCSL) with the nitroxide group at different positions, n = 5,7,10 and 14) in the sn-2 acyl chain, were also purchased from Avanti Polar Lipids. The spin-labels were stored at −20°C in ethanol solutions at a concentration of 1 mg/mL.

#### Sample preparation

Samples of DMPC multi lamellar vesicles (MLV) for ESR spectroscopy were prepared as follows: 20 μg of DMPC, dissolved in a CH_2_Cl_2_–methanol mixture (2:1 v/v), and 1% (wt/wt) of the spin-label, dissolved in ethanol, were thoroughly mixed, and a thin lipid film was produced by evaporating the solvents with dry nitrogen gas. Final traces of solvents were removed by subjecting the sample to vacuum desiccation for at least 3 h. The samples were then hydrated with 20 μL of 10 mM phosphate buffer, gently warmed at ~35°C, and repeatedly vortexed. The lipid suspension thus obtained was transferred into a 25 μL glass capillary.

Samples containing the peptide–lipid complex were prepared in a similar manner, except that the lipid film was hydrated directly with the peptide solution in phosphate buffer. The lipid/peptide molar ratio was set on ~25:1 mol/mol.

#### Electron Spin Resonance measurements

ESR spectra were recorded with a 9 GHz Bruker Elexys E-500 spectrometer (Bruker, Rheinstetten, Germany). Samples were placed in 25 μL glass capillaries and flame sealed. The capillaries were placed in a standard 4 mm quartz sample tube containing light silicone oil for thermal stability. All the measurements were performed at 30°C. Spectra were recorded using the following instrumental settings: sweep width, 100 G; resolution, 1024 points; time constant, 20.48 ms; modulation frequency, 100 kHz; modulation amplitude, 1.0 G; incident power, 6.37 mW. Several scans, typically 16, were accumulated to improve the signal-to-noise ratio. Values of the outer hyperfine splitting, 2A_max_, were determined by measuring, through a home-made MATLAB-based routine, the difference between the low-field maximum and the high-field minimum. This parameter is a useful empirical measure of the lipid chain dynamics and order in both gel and fluid phases of lipid bilayers. The main source of error on the 2A_max_ value is the uncertainty in composition of samples prepared by mixing few microliters of mother solutions. For this reason, reproducibility of 2A_max_ determination was estimated by evaluating its value for selected independently prepared samples with the same nominal composition. The uncertainty affecting the 2A_max_ parameter was ±0.2 G.

### NMR experiments

#### Sample Preparation for NMR analysis

The samples for and NMR experiments in mixed micelles of dodecylphosphocholine(DPC)/(sodium dodecyl sulfate) SDS (90/10 M:M) were prepared by dissolving an appropriate amount of peptide (1.5 mM) in a DPC/SDS water mixture. The DPC concentration used was 27 mM (27 times higher than DPC cmc)^55^, and the molar DPC:SDS ratio was 90/10 (27 mM/3 mM). For NMR experiments, ^d25^SDS and ^d38^DPC were used.

#### NMR analysis

NMR spectra were collected using a Bruker DRX-600 spectrometer at 300 K. One-dimensional (1D) NMR spectra were recorded in the Fourier mode with quadrature detection. The water signal was suppressed by low-power selective irradiation in the homo-gated mode. DQF-COSY, TOCSY, and NOESY^32^,^56^ experiments were run in the phase-sensitive mode using quadrature detection in ω_1_ via time-proportional phase increases of the initial pulse. Data block sizes were 2048 addresses in t_2_ and 512 equidistant t_1_ values. Prior to Fourier transformation, the time domain data matrices were multiplied by shifted sin^2^ functions in both dimensions. A mixing time of 70 ms was used for the TOCSY experiments. NOESY experiments were run with mixing times in the range of 100–300 ms. Qualitative and quantitative analyses of DQF-COSY, TOCSY, and NOESY spectra were achieved using SPARKY software.

#### NMR structure calculations

Peak volumes were translated into upper distance bounds with the CALIBA routine from the CYANA software package^57^. The requisite pseudoatom corrections were applied for non-stereospecifically assigned protons at prochiral centers and for the methyl group. After discarding redundant and duplicated constraints, the final list of experimental constraints was used to generate an ensemble of 100 structures by the standard CYANA protocol of simulated annealing in torsion angle space implemented (using 6000 steps). No dihedral angle or hydrogen bond restraints were applied. Final structures were analyzed using the Insight 98.0 program.

NMR Experiments in the Presence of Spin- Probes were carried out using a solution of 5-Doxyl-stearic acid spin-probes prepared using methanol-*d*4 as solvent. Spin-probes were added to deuterated SDS-*d25* and DPC-*d*38 micellar solutions of CSF114 and CSF114(Glc) in a concentration ratio corresponding to spin probe/micelle 1:1.

## Author Contributions

V.L. and A.M.D'U. designed the work. A.B. and V.L. performed the computational part, M.S. and A.D'U. the NMR experiments and G.D'E. the ESR experiments. A.B., M.S., E.N., G.D'E., A.M.D'U. and V.L. wrote the manuscript.

## Supplementary Material

Supplementary Information Supplementary Information

Supplementary Information CSF(114) binding conformations to membrane

Supplementary Information Binding of CSF114 to membrane

Supplementary Information Binding of CSF114(Glc) to membrane
