# Supplementary material for: The Glycan Role in the Glycopeptide Immunogenicity Revealed by Atomistic Simulations and Spectroscopic Experiments on the Multiple Sclerosis Biomarker CSF114(Glc)
Source: Sci Rep. 2015 Mar 17;5:9200. doi: 10.1038/srep09200 (PMC4361856; doi:10.1038/srep09200)
Supplement: Supplementary Information [file srep09200-s1.pdf]

Supplementary Information

The Glycan Role in the Glycopeptide Immunogenicity Revealed by Atomistic Simulations and Spectroscopic Experiments on the Multiple Sclerosis Biomarker CSF114(Glc)

Agostino Bruno[1,*], Mario Scrima[2,*], Ettore Novellino[1], Gerardino D'Errico[3], Anna Maria D'Ursi[2], Vittorio Limongelli[1,4]

[1]*Department of Pharmacy, University of Naples "Federico II", via D. Montesano, 49, I-80131 Naples, Italy*
[2]*Department of Pharmacy, University of Salerno, Via Ponte don Melillo 11c, I-84084 Fisciano, Italy*
[3]*Dipartimento di Scienze Chimiche, Università di Napoli "Federico II", Complesso di Monte Sant'Angelo, via Cinthia, 80126 Naples, Italy*
[4]*Università della Svizzera Italiana (USI), Faculty of Informatics, Institute of Computational Science, via G. Buffi 13, CH-6900 Lugano, Switzerland.*

1. Supplementary Results

Standard MD Simulations
Supplementary Figure 1 | Glycopeptide/Membrane interactions
Supplementary Figure 2 | RMSD trace for the helix core
Supplementary Table 1 | 2Amax values for pure DMPC bilayer and in presence of CSF114 and CSF114(Glc)
Supplementary Table 2 | TOCSY Spectra in DPC micelles.
Supplementary Figure 3 | Cluster Analysis
Supplementary Figure 4 | CSF114(Glc)-mAb8-18C5 complex

2. Supplementary Methods

Supplementary Figure 4 | Cluster Analysis
Supplementary Figure 5 | FMD setup

Supplementary Table 3 | List of residues considered in the contact collective variable.
Supplementary Figure 6 | Docking Grid Parameters
Setting for the CSF114(Glc) and CSF114 simultations in explicit TIP3P solvent model

3. Supplementary References

1. Supplementary Results

Standard MD simulations of CSF114(Glc) and CSF114. To assess the stability of the binding conformations of CSF114(Glc) and CSF114 identified through FMD calculations, we performed a series of over 100 ns long MD simulations. In particular, the peptides were sampled in their bound state to the membrane (see Fig. 2 in the main text) and in fully solvated systems. Both the peptides show a different behaviour in the diverse environment. In fact, in the membrane environment CSF114(Glc) and CSF114 conserve the α-helix structure throughout all the simulations, showing a good conformational stability particularly in the case of CSF114(Glc) (Supplementary Figure 3). The first binding mode of CSF114(Glc), where the peptide interacts with the phospholipid bilayer through its glycosylated side, is the most stable pose with all the peptide/membrane interactions conserved during the whole simulation (Supplementary Figure 2). We stress that in these simulations the peptides are fully flexible, however in all the cases the α-helix structure is conserved along the over 100 ns MD simulation (Supplementary Figure 2 and Figure 2f and g in the main text).

This structural stability is not present when the peptides are in the bulk water. In this case, the simulations highlight a high conformational flexibility of both the peptides, which lose the starting α-helix structure in the first 10 ns of simulation. This instability is reflected by the high rmsd values computed for CSF114(Glc) and CSF114 during the simulations in fully water systems with respect to the NMR conformations (Supplementary Figure 2). Our findings are in agreement with the experimental observations[1,2] showing that CSF114(Glc) and CSF114 assume random-coil conformations in pure water environment.

Our results demonstrate that the membrane plays a key role in stabilizing the peptides α-helix structure, with the glycosylated peptide overall more stable than the non-glycosylated one (Supplementary Figure 2).

The stability of the two binding conformations of CSF114(Glc), Side 1 and Side 2, has been further evaluated computing the rmsd values of all the Cα atoms with respect to their position in the NMR structure (Figure 2e in the main text). Comparing Figure 2d and 2e in the main text, one can note

that the higher rmsd values reported in Figure 2e are due to the conformational flexibility of the N-ter and C-ter resides and overall the Side 1 binding mode is more stable than the Side 2 one.

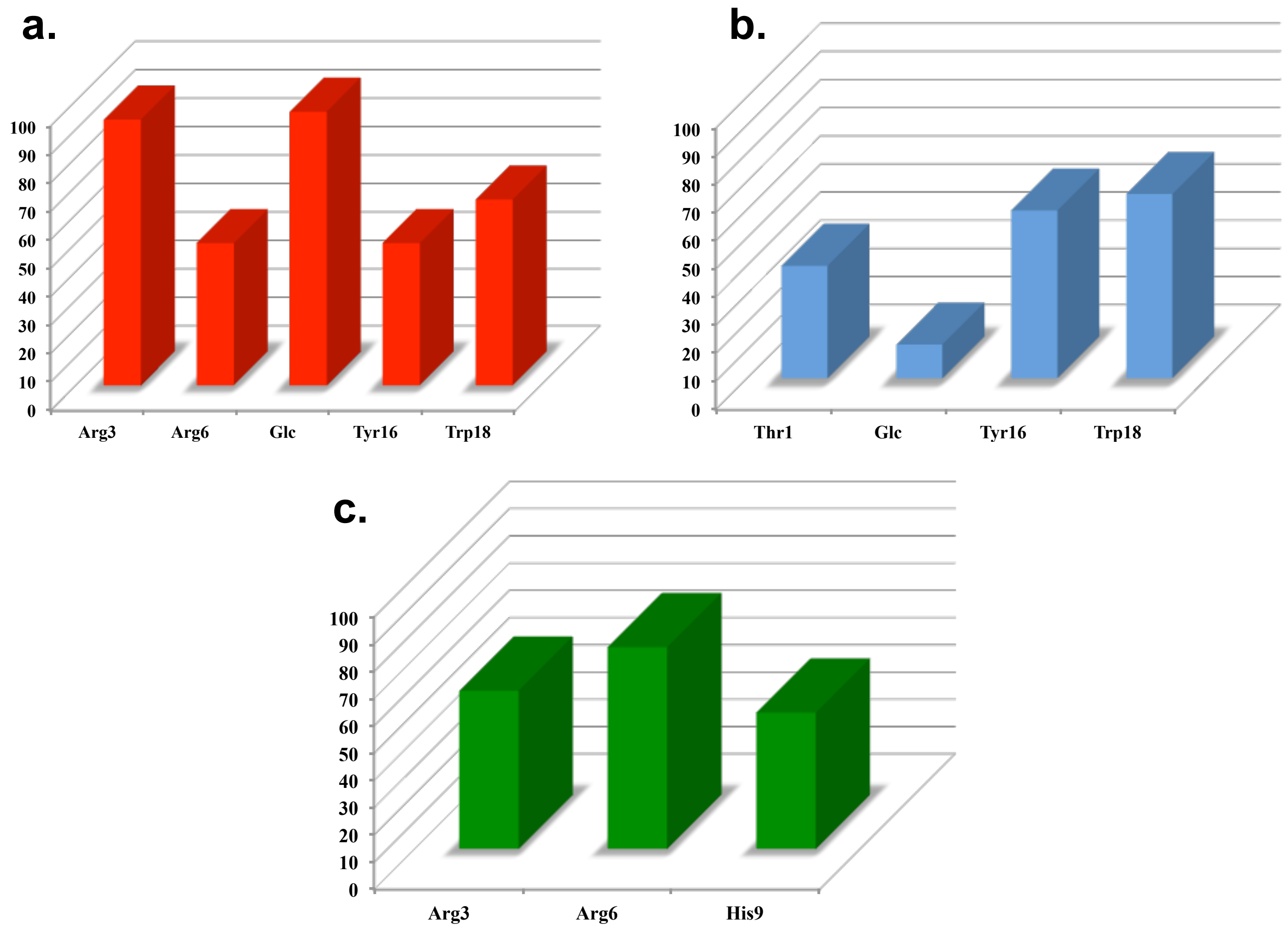

Supplementary Figure 1 | CSF114(Glc)/Membrane and CSF114/Membrane interactions during standard MD simulations. Percentage of the existence of peptide/Membrane interactions along the 100 ns MD simulations for Side 1 (**a**), Side 2 (**b**) of CSF114(Glc), and CSF114 (**c**).

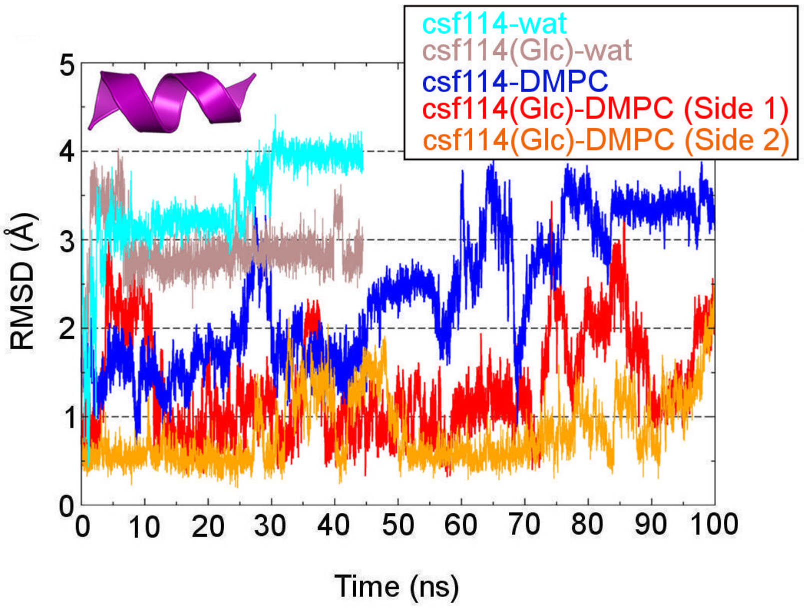

Supplementary Figure 2 | The rmsd plot reveals the α-helix stability. Plot of the rmsd values of the Cα atoms of the core residues forming the α-helix structure (5-12 aa) computed with respect to the NMR conformation.

Supplementary Table 1 | 2Amax values for pure DMPC bilayer and in presence of CSF114 and CSF114(Glc). It is possible to note that, in all cases, the flexibility gradient with the chain position of the lipid bilayer membranes is preserved. However, inspection of Table 1 reveals a significantly different behavior of the lipid chain mobility depending on the presence of the two peptides.

$2A_{max}$ /G				
**Probe Position**	**5-PCSL**	**7-PCSL**	**10-PCSL**	**14-PCSL**
**DMPC**	49.8 ± 0.1	46.1 ± 0.1	43.2 ± 0.1	33.6 ± 0.1
**+ CSF notGlyco peptide**	50.6 ± 0.2	46.4 ± 0.2	42.9 ± 0.1	33.6 ± 0.2
**+ CSF Glyco peptide**	46.3 ± 0.1	41.2 ± 0.1	35.1 ± 0.2	32.8 ± 0.1

Supplementary Table 2 | TOCSY spectra in DPC micelles. Attenuation of the NH signals in the TOCSY Spectra of CSF114(Glc) and CSF in 50mM of DPC by spin-labels 5-DS**.**

**Residue**	**CSF114(Glc) +5-DS**	**CSF114+5-DS**
**THR 1**	Strong	Strong
**PRO 2**	/	/
**ARG 3**	No	No
**VAL 4**	Medium	Strong
**GLU 5**	Strong	Strong
**ARG 6**	No	No
**ASN 7**	Medium	No
**GLY 8**	No	No
**HIS 9**	No	No
**SER 10**	No	No
**VAL 11**	Medium	Medium
**PHE 12**	Medium	Strong
**LEU 13**	Strong	Strong
**ALA 14**	Strong	Strong
**PRO 15**	/	/
**TYR 16**	Strong	Strong
**GLY 17**	Strong	Strong
**TRP 18**	Strong	Strong
**MET 19**	Strong	Strong
**VAL 20**	Strong	Strong
**LYS 21**	Strong	Strong

Strong (>70%), Medium (30-70%) and No or / (0%)

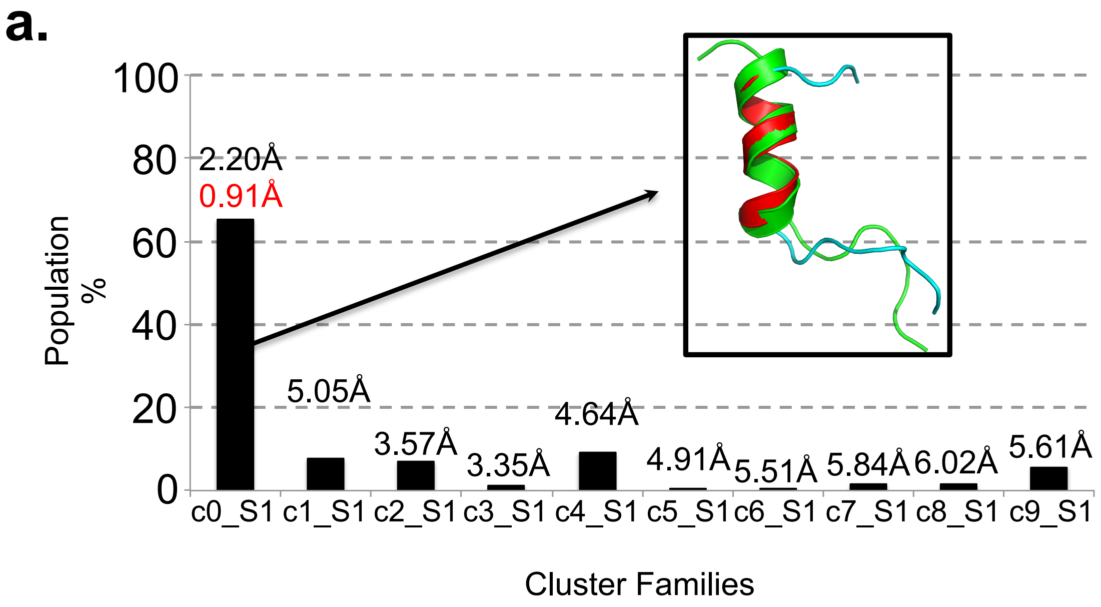

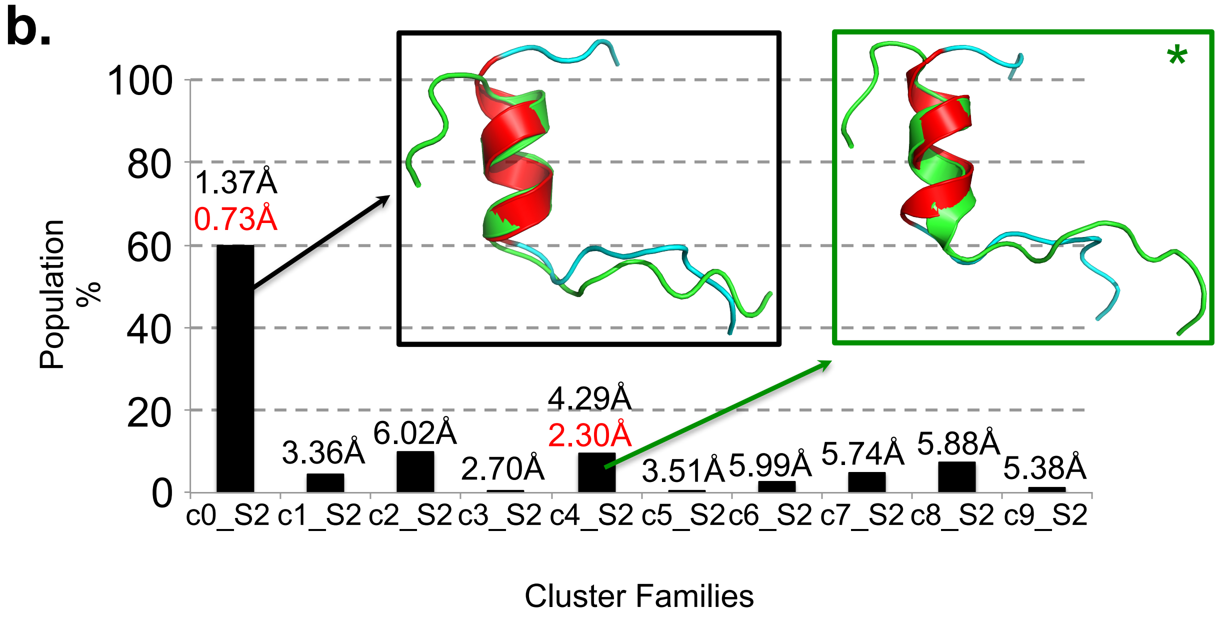

Supplementary Figure 3 | Cluster analysis of standard MD simulations. a. Results of the cluster analysis of the 100 ns long MD simulation of the Side 1 binding mode of CSF114(Glc) (see Fig. 2c in the main text). In the black box the structure with the lowest rmsd value with respect to the NMR structure is reported; **b.** Results of the cluster analysis of the 100 ns long MD simulation of the Side 2 binding mode of CSF114(Glc) (see Fig. 2d in the main text). In the black box the structure with the lowest rmsd value with respect to the NMR structure is reported, while in the green box the structure that gave the best docking result. In black above the bars are reported the rmsd values computed for all the Cα atoms of the representative conformation of each family with respect to the NMR structure, while in red are the rmsd values computed only for the Cα atoms forming the α-helix core (5-12 aa).

The representative conformation of each family was undergone to docking calculations.

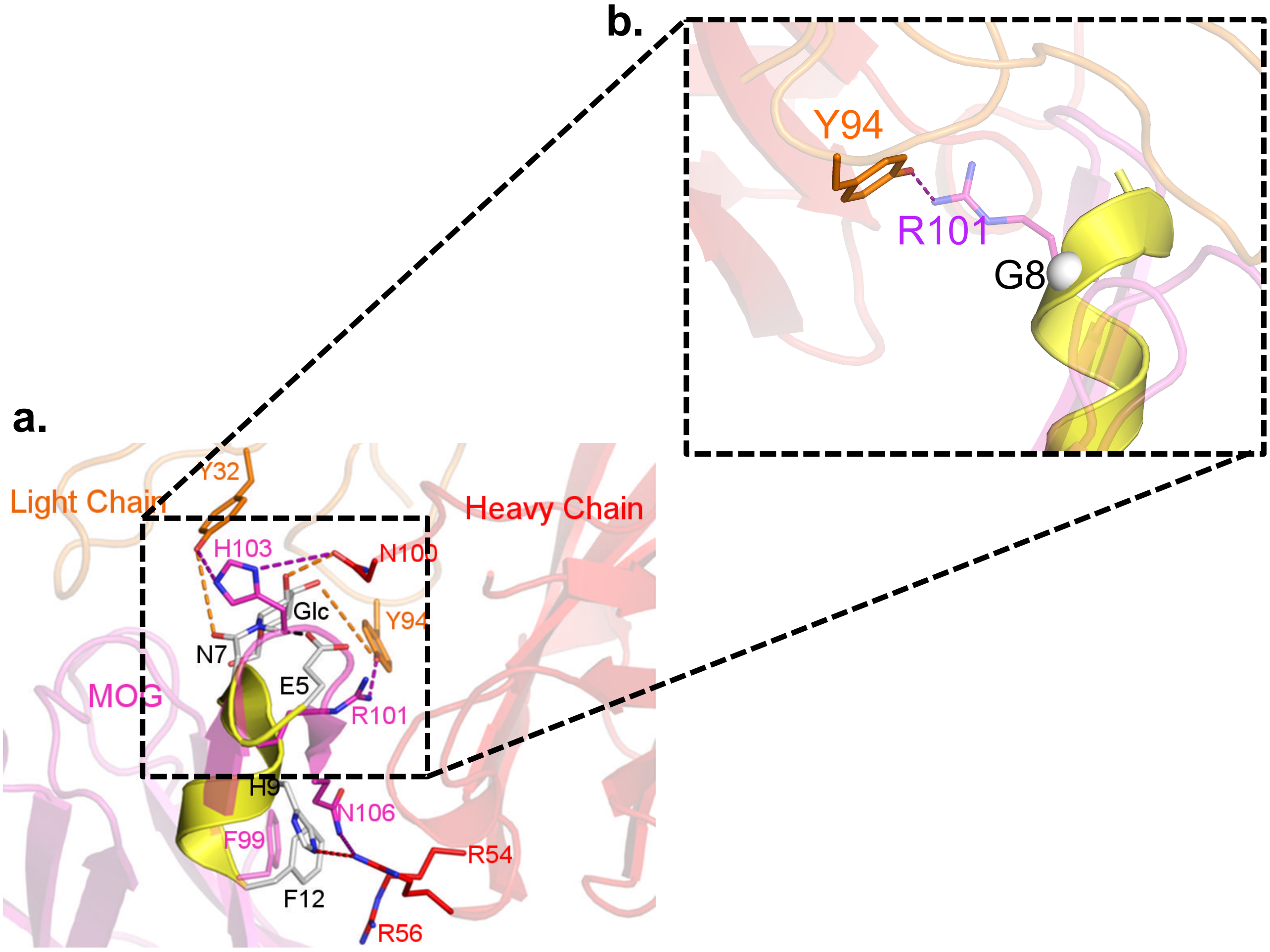

Supplementary Figure 4 | Hints for probe design. a. Superimposition of the computed binding mode of CSF114(Glc) on the X-ray crystal structure of the MOG/mAb8-18C5 complex. The CSF114(Glc) peptide is shown in yellow with white stickes, the light and heavy chains of autoAb are colored in orange and red, respectively, and the MOG is represented in purple; **b.** The inset shows the position of Gly8 (Cα as white sphere) in the CSF114(Glc)/mAb8-18C5 complex, which might be mutated in a residue able to mimic the interaction engaged by Arg101 of MOG (purple sticks) with Tyr94 of mAb8-18C5 (orange sticks).

2. Supplementary Material and Methods

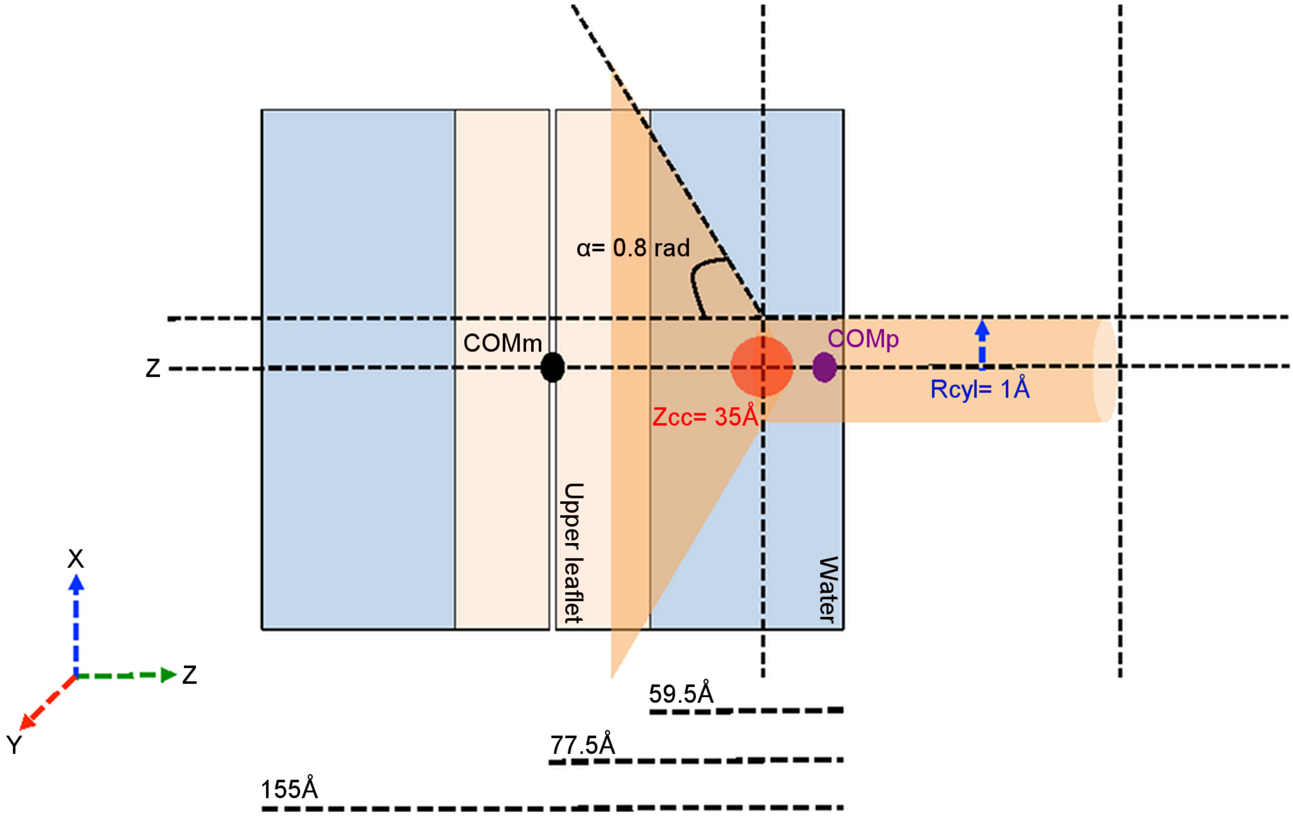

Supplementary Figure 5 | FMD setup. Schematic representation of the funnel restraint potential used in FMD calculations. COMm= center of mass of the membrane; COMp= center of the mass of the peptide; Z is the axis perpendicular to the membrane surface; Zcc is the distance from COMm where the restraint potential switches from a cone shape into a cylinder; the α-angle (rad) defines the amplitude of the cone; Rcyl is the radius of the cylindrical section. We stress that inside the funnel the external bias felt by the system is equal to 0, while a repulsive potential, felt by the system when COMp reaches the edge of the funnel, discourages the system to visit regions outside the funnel (see ref. 25 in the main text for details).

Supplementary Table 3 | List of residues considered in the contact collective variable. List of the residues considered for the contact collective variable for both CSF114(Glc) and CSF114. The protein Cα atoms and the phosphate groups of the membrane were considered to compute the contact collective variable values.

Side 1	Side 2
**Arg6**	Glu5
**Asn7**	His9
**Ser10**	Leu13
**Val11**	Phe12

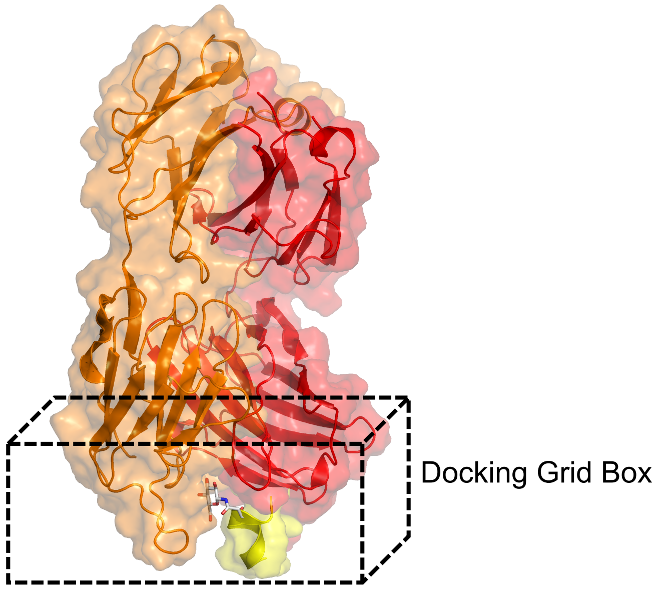

Supplementary Figure 6 | Docking grid box. Docking calculations between CSF114(Glc) and mAb8-18C5 were performed in the region defined by the grid box (dashed lines), which was centered on the complementary-determining regions (CDRs) of the light and heavy chains where antigen/antibody binding typically occurs.

Setup of the CSF114(Glc) and CSF114 MD simulations in fully water systems.

System Parametrization. The CSF114(Glc) was obtained from the NMR experiments described in the main text and then parameterized using the *ff99SBildn*, *gaff*, and *GLYCAM_06* force fields of Amber11 suite[3]. The structure of the non-glycosylated peptide, CSF114, was obtained from the NMR experiments described in the main text and then parameterized using the *ff99SBildn* force field.

MD simulations. All the simulations were carried out using the NAMD 2.8 software[4,5] and TIP3P as water solvation model[6]. The net charge of each system was neutralized adding 2 Cl- ions. The initial box dimension was 51.60 x 50.00 x 60.50 Å for both peptides. The CSF114(Glc)-TIP3P and CSF114-TIP3P system consist of 14192 and 11999 atoms, respectively. The equilibration phase was performed as follows: (*i*) 4000 steps of conjugate gradient minimization, where harmonic constraints on the system were progressively reduces; (*ii*) 90 ps of heating phase from 50 K to 300 K using the Langevin thermostat, where harmonic constraints on the peptide backbone atoms were progressively reduces; (*iii*) 70 ps of pressure equilibration with a target pressure of 1.01325 bar and using the Nosè-Hoover Langevin barostat, where harmonic constraints were gradually switched off; (*iv*) 100 ps of pressure equilibration without constraints; (*v*) 100 ns of production phase. The production phase was carried out using the periodic boundary conditions in the NVT ensemble. The time-step was set to 2 fs and the RATTLE algorithm was used for the hydrogen atoms. The temperature was retained at 300 K using the Langevin thermostat. All the simulations were conducted using a cutoff of 12 Å for both electrostatic and van der Walls interactions, while the long-range electrostatics were treated using the PME methodology[7] with a grid spacing of 1.0 Å and an interpolation order of 4.

Supplementary References

1. Lolli, F. *et al.* An N-glucosylated peptide detecting disease-specific autoantibodies, biomarkers of multiple sclerosis. *Proc. Natl. Acad. Sci. U. S. A.* **102,** 10273–10278 (2005).
2. Pandey, S. *et al.* Designed Glucopeptides Mimetics of Myelin Protein Epitopes As Synthetic Probes for the Detection of Autoantibodies, Biomarkers of Multiple Sclerosis. *J. Med. Chem.* **55,** 10437–10447 (2012).
3. The Amber Molecular Dynamics Package. Available at: http://ambermd.org/
4. Phillips, J. C. *et al.* Scalable molecular dynamics with NAMD. *J. Comput. Chem.* **26,** 1781–1802 (2005).
5. NAMD - Scalable Molecular Dynamics. Available at: http://www.ks.uiuc.edu/Research/namd/
6. Lamoureux, G., Harder, E., Vorobyov, I. V., Roux, B. & MacKerell, A. D. A polarizable model of water for molecular dynamics simulations of biomolecules. *Chem. Phys. Lett.* **418,** 245–249 (2006).
7. Darden, T., York, D. & Pedersen, L. Particle mesh Ewald: An N·log(N) method for Ewald sums in large systems. *J. Chem. Phys.* **98,** 10089 (1993).
